# Development of 3D *in Vitro* Technology for Medical Applications

**DOI:** 10.3390/ijms151017938

**Published:** 2014-10-08

**Authors:** Keng-Liang Ou, Hossein Hosseinkhani

**Affiliations:** 1 Nanomedicine Research Center of Taiwan, College of Oral Medicine, Taipei Medical University, Taipei 110, Taiwan; E-Mail: klou@tmu.edu.tw; 2 Research Center for Biomedical Devices and Prototyping Production, College of Oral Medicine, Taipei Medical University, Taipei 110, Taiwan; 3 Research Center for Biomedical Implants and Microsurgery Devices, College of Oral Medicine, Taipei Medical University, Taipei 110, Taiwan; 4 Graduate Institute of Biomedical Materials and Engineering, College of Oral Medicine, Taipei Medical University, Taipei 110, Taiwan; 5 Department of Dentistry, Taipei Medical University-Shuang-Ho Hospital, Taipei 110, Taiwan; 6 Graduate Institute of Biomedical Engineering, National Taiwan University of Science and Technology, Taipei 10607, Taiwan

**Keywords:** biomaterials, tissue engineering, 3D *in vitro*, polymer, hydrogel

## Abstract

In the past few years, biomaterials technologies together with significant efforts on developing biology have revolutionized the process of engineered materials. Three dimensional (3D) *in vitro* technology aims to develop set of tools that are simple, inexpensive, portable and robust that could be commercialized and used in various fields of biomedical sciences such as drug discovery, diagnostic tools, and therapeutic approaches in regenerative medicine. The proliferation of cells in the 3D scaffold needs an oxygen and nutrition supply. 3D scaffold materials should provide such an environment for cells living in close proximity. 3D scaffolds that are able to regenerate or restore tissue and/or organs have begun to revolutionize medicine and biomedical science. Scaffolds have been used to support and promote the regeneration of tissues. Different processing techniques have been developed to design and fabricate three dimensional scaffolds for tissue engineering implants. Throughout the chapters we discuss in this review, we inform the reader about the potential applications of different 3D *in vitro* systems that can be applied for fabricating a wider range of novel biomaterials for use in tissue engineering.

## 1. Introduction

Over the past decade, the development of biomaterials and tissue engineering technology has stimulated great interest in the possibility of creating three dimensional (3D) *in vitro* models to better understand the mechanism behind cellular fate. 3D *in vitro* technology is very interdisciplinary in nature and brings together the field of polymer chemistry, pharmaceutical science, biology, and basic and clinical medicines. The elucidation of using 3D *in vitro* systems will open many doors to significantly improve the quality of biological tools and lead identification as well as therapeutic approaches. Especially in the case of human cells, it may be of clinical relevance for future cell-based therapeutic applications. Also, it will provide attractive combinational strategy of tissue engineering principles with materials engineering to accelerate and enhance tissue regeneration.

**Figure 1:**
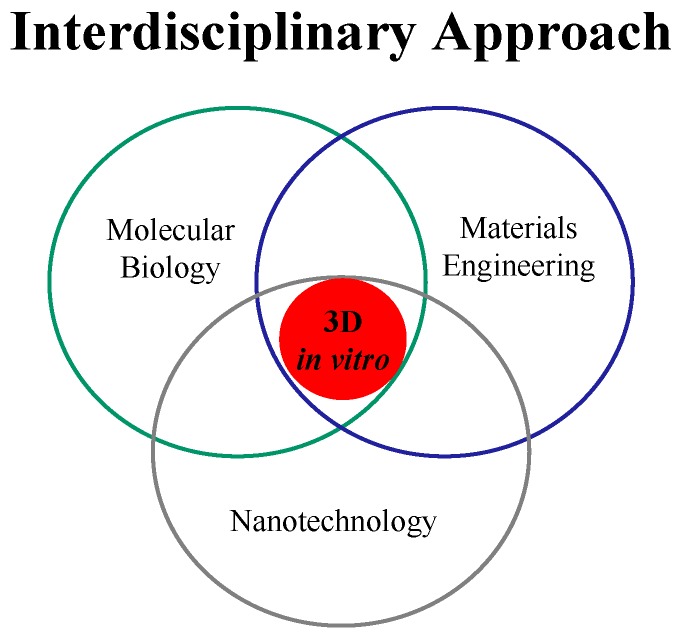
Interdisciplinary approach of 3D *in vitro* technology.

It covers a wide range of applications, including: Drug discovery; micro- and nano-engineering; cellular microenvironment; biomaterials; and high-throughput technologies. Irrespective of the final goal for experimental biology and clinical medicine, the first key issue to be dealt is to engineer cellular microenvironment 3D models as efficiently as possible and to facilitate an *in vivo*-like condition. Combinational technology of materials science and engineering with biology is expected to enhance the quality of valuable biological, pharmaceutical and food products. However, the idea underlying 3D *in vitro* models, that cellular microenvironment might be mimicked by combinational technology of materials science and biology, rather than by conventional technology, has yet to make its mark in clinical medicine. The concept may appear to be elegantly straightforward and the most direct application of 3D technology must be in the biological field. Recent researches have indicated that successful implementation of 3D *in vitro* models in clinic will require the coordinated development of a variety of new technologies and the establishment of unique interactions between investigators from divergent medical and basic science disciplines. Many 3D models that are currently in practice, however, require expensive equipment, large sample volumes, long incubation times and/or extensive expertise, and the most disadvantages of them is that they are too far from the nature of human organs. Because of the above problems, research and development on drug discovery, regenerative medicine, biotech and pharmaceutical Industries are very costly and take several years to bring a single drug/product to the marketing. 3D *in vitro* technology is an interdisciplinary approach to merge biomaterials and tissue engineering science, nanotechnology, and biological principles to generate a platform technology, the so-called 3D living systems to mimic organ/tissues in order to partially reduce the amount of *in vitro* and *in vivo* animal testing, clinical trials, and to solve the above problems (Figure 1).

This review will overview the concept of 3D *in vitro* technology with different systems and suggests new areas of investigation that may help to resolve them.

## 2. Three Dimensional (3D) Engineered Biomaterials

### 2.1. Microscale Biomaterials

Different processing techniques have been developed to design and fabricate 3D microscale scaffolding biomaterials for tissue engineering implants. Tissue engineering needs 3D scaffolds to serve as a substrate for seeding cells and as a physical support in order to guide the formation of the new tissue The majority of the used techniques utilize 3D polymeric scaffolds, which are composed of natural or synthetic polymers. Synthetic materials are attractive because their chemical and physical properties (e.g., porosity, mechanical strength) can be specifically optimized for a particular application. The polymeric scaffolds structures are endowed with a complex internal architecture, channels and porosity that provide sites for cell attachment and maintenance of differentiated function without hindering proliferation. Ideally, a polymeric scaffold for tissue engineering should have the following characteristics: (1) To have appropriate surface properties promoting cell adhesion, proliferation and differentiation; (2) To be biocompatible; (3) To be highly porous, with a high surface area/volume ratio, with an interconnected pore network for cell growth and flow transport of nutrients and metabolic waste; and (4) To have mechanical properties sufficient to whistand any *in vivo* stresses [1]. The last requisite is difficult to combine with the high porosity in volume of the material. That is why it is necessary to use polymeric matrices with special or reinforced properties, especially if the polymer is a hydrogel. The polymeric scaffold design depends on the regarded applications, but in any case it must achieve structures with the aforementioned characteristics, which are necessary for their correct function. To achieve this with success is conditional on two factors: materials used, both the porogen, and the reticulate polymer, which is infiltrated in the porogen to become a scaffold; and, as a second factor, the structural architecture, both external and internal, basically shown by its porosity (high surface area/volume ratio), geometry, size pore and having in mind that the structures must be easily processed into three-dimensional. On the basis of the extensive range of polymeric materials, different processing techniques have been developed to design and fabricate 3D scaffolds for tissue engineering [2].

Hydrogels have attracted great interest as scaffolding materials for 3D *in vitro* technology because of their high water content, biocompatibility and mechanical properties, which resemble those of natural tissues [3,4,5,6]. Hydrogels have been used for tissue engineering of bone, cartilage, vascular and other tissues. By adding cells to a hydrogel precursor prior to the gelling process, cells can be distributed homogeneously throughout the gel. In addition, hydrogels can be used to deliver soluble or immobilized signaling molecules to cells, act as support structures for cell growth and function, and provide space filling for future tissue ingrowth. For example, growth factors, such as transforming growth factor β (TGF-β) have been tethered to poly(ethylene glycol) (PEG) hydrogels to regulate smooth muscle cell function and bone morphogenetic protein (BMP-2) has been covalently attached to alginate to regulate osteoblast migration and calcification into the gels. Also, differentiated cardiac tissues have been engineered by casting neonatal rat cardiac myocytes into collagen gels and subsequently subjecting them to cyclic mechanical stretch. Gelatin has been widely used as 3D hydrogel for several applications in tissue engineering. Gelatin is biodegradable denatured collagen and has been extensively utilized for pharmaceutical and medical purposes. Its biosafety has been proven through long clinical applications [7]. In general, hydrogels from natural sources can be derived from polymers such as collagen, hyaluronic acid (HA), fibrin, alginate, agarose or chitosan. Depending on their origin and composition, various natural polymers have specific utilities and properties. Many natural polymers, such as collagen, HA and fibrin, are derived from various components of the mammalian extracellular matrix (ECM). The advantages of natural polymers include low toxicity and biocompatibility. Collagen and other mammalian derived protein-based polymers are effective matrices for cellular growth, as they contain many cell signaling domains present in the *in vivo* ECM. Collagen gels can be naturally created without chemical modifications. However, in many cases these gels are mechanically weak. To synthesize gels with enhanced mechanical properties various methods have been developed such as chemical crosslinking, UV crosslinking or in mixture with other polymeric agents. Collagen degradation is mediated naturally by proteins such as collagenase. The most abundant heteropolysaccharides in the body are the glycosaminoglycans (GAGs). They are long unbranched polysaccharides containing a repeating disaccharide unit that contains either of two modified sugars: *N*-acetylgalactosamine or *N*-acetylglucosamine and an uronic acid such as glucoronate or iduronate. GAGs are located primarily on the surface of cells or in the ECM. HA is a GAG which is particularly prevalent during wound healing and in joints. Covalently crosslinked HA hydrogels can be formed by multiple chemical modification means. HA can be degraded by cells through release of enzymes such as hyaluronidase. HA is particularly appealing for tissue engineering as it is naturally present in great abundance in a variety of tissues. Previously, HA scaffolds have been used for tissue engineering of various tissues. In addition, composite HA-PEG scaffolds have been used for tissue engineering.

### 2.2. Nanoscale Biomaterials

Nanoscale materials may provide a suitable material for tissue engineering since they can be used to enhance cell adhesion. In particular, the diameters of fibers play an important role in the adhesion of the cells to the fibers. Takahashi *et al.*, compared the attachment of mesenchymal stem cells on microscale non-woven polythylene terephalat (PET) fibers prepared with different diameters [8]. Many biodegradable synthetic polymers, such as PGA and its copolymers with l-lactic acid, d, l-lactic acid, and ε-caprolactone have been fabricated into 3D nanofibers sheets for tissue engineering applications [8]. Furthermore, it has been demonstrated that polyaniline (PANi) and poly (d,l-lactide-*co*-glycolide) (PLGA) fibers that ranged in diameter from 500 to 800 nm enhanced cell adhesion [9]. Also, poly(ε-caprolactone) nanofibers (diameter = 700 nm) were shown to be a suitable carrier for mesenchymal stem cells transplantation [10]. However, it is not yet clear how the diameter of the fibers affect tissue engineering scaffolds for skin regeneration.

The design of materials that can regulate cell behavior such as proliferation and differentiation is a key component for the fabrication of tissue engineering scaffolds. From the viewpoint of immune system response of the body, the implanted biomaterials should mimic the structure and biological function of native ECM, both in terms of chemical composition and physical structure as reported by Ma *et al* [11]. Therefore, in order to mimic the biological function of ECM proteins, the scaffold materials used in tissue engineering need to be chemically functionalized to promote tissue regeneration as ECM does. Collagen and elastin as ECM proteins are made from fibers in dimension smaller than micrometers. It seems that artificial nanoscaled fibers have great potential application in the field of biomaterials and tissue engineering.

The initial report showed that nanoscaled features influenced cell behaviors [12]. Nanoscaled surface topography has been found to promote osteoblast adhesions [11]. It has been demonstrated that osteoblast adhesion, proliferation, alkaline phosphatase activity, and ECM secretion on carbon nanofibers increased by decreasing fiber diameter in the range of 60–200 nm, whereas the adhesion of other kinds of cells such as chondorocytes, fibroblasts, and smooth muscle cells was not influenced [13]. It has been supposed that the nanoscaled surface affects the conformation of adsorbed adhesion proteins such as vitronectin, thus affecting the cell behaviors [14]. In addition, the nanoscaled dimensions of cell membrane receptors such as integrins should also be considered. There are three different approaches toward the formation of nanofibrous materials; phase separation, electrospinning and self-assembly [15]. Phase separation and self-assembling of biomolecules can generate smaller diameter nanofibers in the same range of natural ECM, while electrospinning generate large diameter nanofibers on the upper end of the range of natural ECM [16]. Electrospinning is a common technique used to fabricate nanofibers sheets to be used as 3D scaffolds for tissue engineering applications. It is easy technique and cheap and can be applied for many different types of polymers. Recent study demonstrated that fabricated PGA/collagen nanofibers through electrospinning significantly enhanced cell adhesion compared with PGA/collagen microfibers [17]. It has been reported that this electrospun scaffolds can be used to generate aligned nanofibers (300 nm) of poly l-lactic acid (PLLA), which improve the differentiation of neural stem cells (NSC) and support neuronal outgrowth in comparison to fibers with larger diameters [18]. Therefore, the availability of aligned nanofibers can be potentially useful in tissue engineering. The adhesion of NIH-3T3 fibroblasts has been studied on poly(p-dioxanone-*co*-l-lactide)-block-poly (ethylene glycol) (PPDO/PLLA-b-PEG) nanofibers [12]. However, there are no studies on the interaction of individual cells and nanofibers in a systematic manner. To study the mechanism of interaction between cells and nanofibers, it is important to control the architecture of nanofibers to minimize the interaction of a single cell with the neighboring fibers. Therefore, aligned micro- and nanofibers in which the adherent cells only interact with individual fibers is a potentially useful approach for studies that aim to analyze such interactions. However, it is not yet clear factors affect on cell adhesion between materials and extracellular matrix (ECM) proteins [19]. To study the effect of fiber diameter on the adhesion of fibroblast cells, Tian *et al.*, set up a quantitative electrospinning model [20]. In their study, they used a number of biomaterials that are commonly used in tissue engineering and were natural components of the cellular microenvironment. Specifically, they used poly glycolic acid (PGA) a well-known biomaterial, and collagen which is one of the main components of the ECM. Electrospun composite nanofibers comprising of various ratios of PGA and collagen were fabricated with diameters in the range of 3–5, 10 and 500 nm. They analyzed the properties of NIH-3T3 cells on these fibers as a function of fiber diameter and structure. It was demonstrated that the fiber composition and diameter had a direct influence on the morphology and alignment of the fibroblast cells, indicating that fiber properties could be used for engineering scaffolds that induced specific functional properties on seeded cells.

One of the common approaches to produce fibers similar to ECM proteins such as collagen is self-assembly [21,22]. Specific peptide containing 16 alternating hydrophobic and hydrophilic amino acids was fabricated to self-assemble into nanofibers under appropriate pH values [23]. Nanoscaled fibers produced by self assembly of amphiphilic peptide may be a promising approach in designing the next generation of biomaterials for drug delivery and tissue engineering [23,24]. It would be beneficial for biomedical applications if scaffold materials could promote the adhesion and growth of cells on their surfaces. The sequences of arginine-glycine-aspartic acid (RGD) has been discovered as a cell attachment sequence in various adhesive proteins present in the ECM, and found in many proteins, such as fibronectin, collagen type 1, vitronectin, fibrin, and von willebrand factor. It has been recognized that the sequence of RGD interacts with various types of integrin receptors of mammalian cells. Ever since the RGD sequence has been discovered as a cell attachment sequence in adhesive proteins of the ECM, there have been several efforts to synthesize bioactive peptides incorporating RGD for therapeutic purpose. Micro-and nanopatterned scaffolds have been investigated less well in regard to stem cells, although two recent studies highlight their attractiveness. It is hypothesized that the density of the cells-binding ligands to which the cells are exposed indicates clearly the importance of extracellular matrix in influencing cell function. Recent studies have indicated that when the Isolucine–Lysine–Valine–Alanine–Valine (IKVAV) mimicking laminin-specific domain was replaced with the amino acid sequence, arginine-glycine-asparate (RGD), a common cell-binding domain in many extrecellular matrix proteins, especially collagen, differentiation of dorsal root ganglion (DRG) to neural cells was significantly enhanced compared with those without this sequence or to two-dimensional controls [25]. The proliferation of cells in the 3D scaffold needs an oxygen and nutrition supply. In this circumstance, the 3D scaffold materials should provide such an environment for cells. The artificial scaffolds formed by self-assembling molecules not only provides a suitable support for cell proliferation but also serves as a medium through which diffusion of soluble factors and migration of cells can occur. The result of the cell attachment and proliferation revealed that diffusion of nutrients, bioactive factors, and oxygen through these highly hydrated networks is sufficient for survival of large numbers of cells for extended periods of time.

## 3. 3D Cellular Microenvironment

### 3.1. Extracellular Matrix

**Figure 2:**
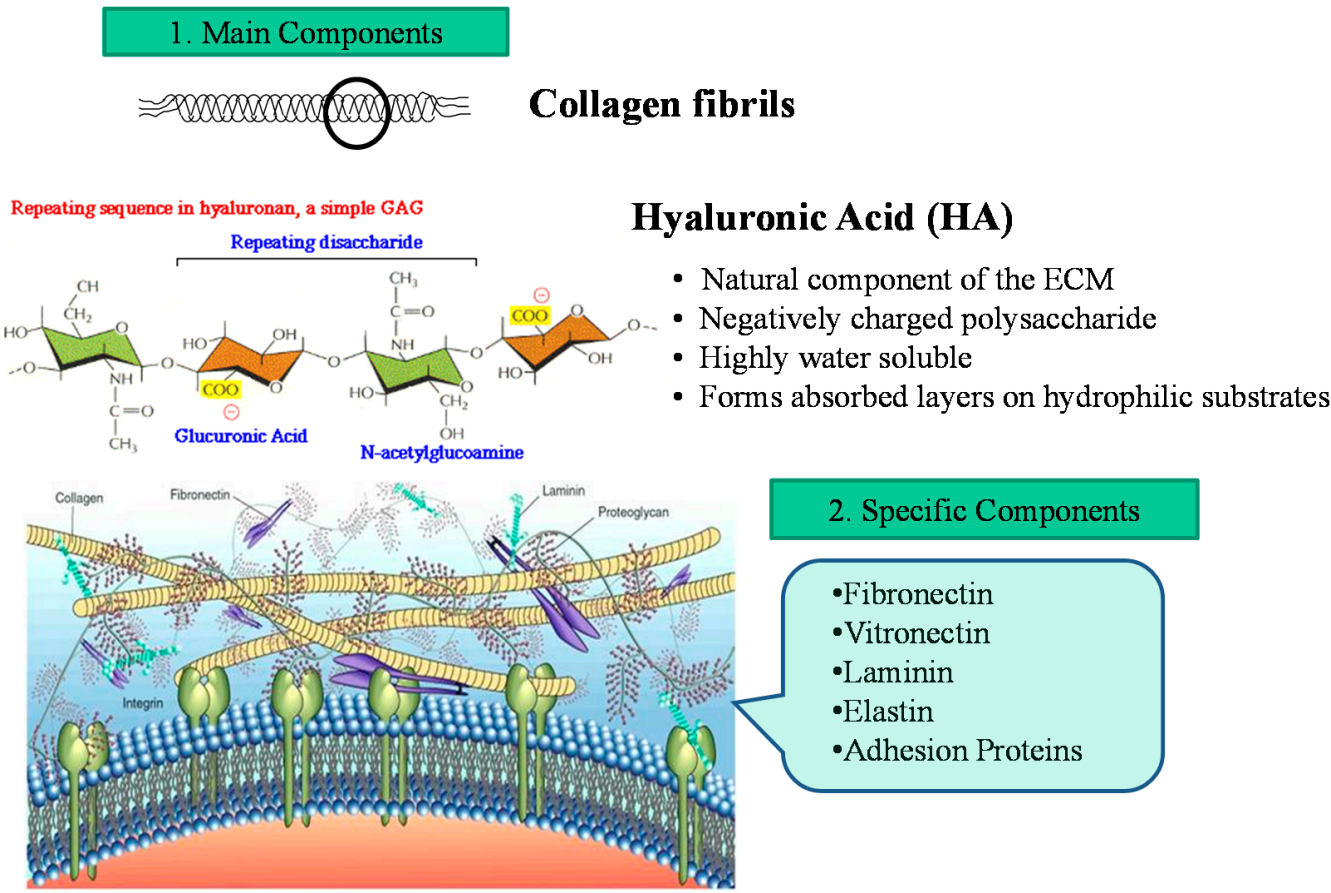
Cellular microenvironment showing main and specific components of extracellular matrix (ECM).

The extracellular matrix (ECM) is the extracellular part of animal tissue that usually provides structural support to the animal cells in addition to performing various other important functions. The extracellular matrix is the defining feature of connective tissue in animals. Extracellular matrix includes the interstitial matrix and the basement membrane. Interstitial matrix is present between various animal cells (*i.e.*, in the intercellular spaces). Gels of polysaccharides and fibrous proteins fill the interstitial space and act as a compression buffer against the stress placed on the ECM. Basement membranes are sheet-like depositions of ECM on which various epithelial cells rest. Due to its diverse nature and composition, the ECM can serve many functions, such as providing support, segregating tissues from one another, and regulating intercellular communication. The ECM regulates a cell’s dynamic behavior. In addition, it sequesters a wide range of cellular growth factors, and acts as a local depot for them. Changes in physiological conditions can trigger protease activities that cause local release of such depots. This allows the rapid and local growth factor-mediated activation of cellular functions, without *de novo* synthesis. Formation of the extracellular matrix is essential for processes like growth, wound healing and fibrosis. An understanding of ECM structure and composition also helps in comprehending the complex dynamics of tumor invasion and metastasis in cancer biology as metastasis often involves the destruction of extracellular matrix by enzymes such as serine and threonine proteases and matrix metalloproteinases. Figure 2 shows schematic illustration of ECM. The main components of ECM are Proteoglycans, Heparan sulfate, Chondroitin sulfate, Keratan sulfate. Also Non-proteoglycan polysaccharides are involved in ECM. They are included: Collagen, elastin, fibronectin, and laminin.

Since cells produce ECM proteins such as fibronectin and collagen that is commonly found in most organs and is not present in the brain; therefore, biomaterials should be selected based on the specific tissue/organ that is engineered [26,27].

Several 3D models are developed without scaffolds as the cells produce their own ECM. When choosing a scaffolding material, several factors such as scaffold-mimic the cells and native extracellular matrix, formation of functional multicellular structures, and imaging the cells are considered. It can be challenging to image cells grown on some scaffolds. Spheroids are self-assembled spherical clusters of cell colonies. They were first documented in 1944 by Johannes Holtfreter who worked with spherical aggregates of embryonic cells. Spheroids naturally mimic solid tissues, avascular tumors, and embryoid bodies, and have found application among researchers in cancer and stem cell research. With inherent metabolic (oxygen, carbon dioxide, nutrients, wastes) and proliferative gradients, spheroids serve as excellent physiologic models. Scaffold-free platforms for spheroid growth do not contain added biomaterials or ECM, and cells grown in them generate and organize their own 3D ECM, so spheroids closely resemble *in vivo* tissues. Co-cultures with other cell types (*i.e.*, endothelial, stromal, epithelial cells) extend the predictive cytotoxicity capabilities of this 3D cell culture system. Scaffold-free platforms have no support structure or porosity. The overall spheroid size is limited beyond a critical size of 500–600 µm in diameter, after which central secondary necrosis develops in most, but not all, spheroids grown from permanently-transformed cell lines.

### 3.2. Cellular Microenvironment (Niche)

The cellular microenvironment/niche plays a significant role in the regulation of a host of physiological and pathophysiological processes. The ability to control the cellular microenvironment is key to controlling cell viability, growth, migration, apoptosis, and differentiation. Niche refers to a microenvironment in which a cell is situated. Thus, a stem cell niche is a microenvironment surrounding a stem cell. In addition to possible contributors to the stem cell niche, the microenvironments also comprised of many ECM and signaling molecules. Signaling molecules are molecules that are entrapped inside the ECM. LaBarge and Bissell *et al.* found that adult human mammary stem and progenitor cells exhibit impressive plasticity in response to hundreds of unique combinatorial microenvironments [28]. They also suggested that rational modulation of the micro-environmental milieu can impose specific differentiation phenotypes on normal stem or progenitor cells, and perhaps even impose phenotypically normal behavior on malignant cells during tissue genesis. All of this points to the rational manipulation of adult stem and progenitor cells as a promising pathway for beneficial therapies. The ability of adult stem cells to self-maintain, as well as to give rise to progenitor cells that are targeted to become a specific tissue cell, indicates an ability to respond to changing micro-environmental demands, which would mean that a stem or progenitor cell is receiving instructional information from its surroundings. Microenvironment microarrays (MEArrays) technology developed by LaBarge and Bissell can expose stem cells to many different proteins and biological molecules simultaneously. This enables researchers to mimic in cell culture studies the complex microenvironments that determine the ultimate fate of a stem or progenitor cell in a living organism.

**Figure 3:**
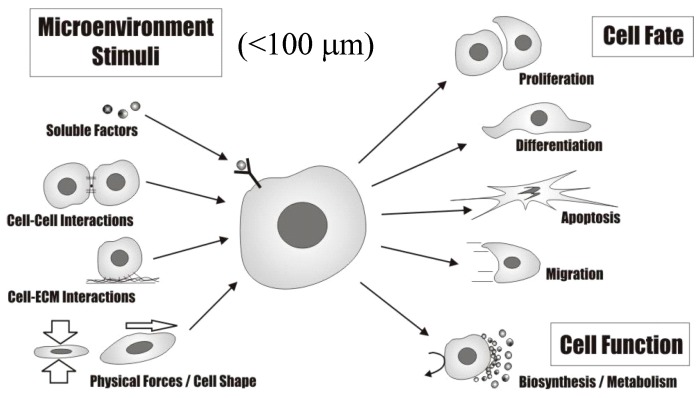
The cell microenvironment (niche) directs cellular fate and function.

An organ is a group of tissues that perform a function, as well as the associated blood vessels and extracellular matrix. A group of organs is called a system. For example, the heart, together with all the blood vessels in the body, is part of the cardiovascular system. The heart is made up of a main tissue called the myocardium. It also has connective tissues, nerves, and blood vessels. The bone also contains several different types of tissues: compact bone, spongy bone, and bone marrow. A tissue is a group of connected cells that perform the same function. The four main types of tissue are: Epithelium tissue—Layers of cells that line surfaces in the body. Examples include the skin and the inner lining of the gut. Connective tissue—It connects other tissues. This is the tissue that contains the extracellular matrix. Examples include bone and blood. Muscle tissue—Muscles are what cause our bodies to move: either through the world spatially, such as walking, or in the body internally, like the beat of your heart or the peristaltic movements of your esophagus. Nervous tissue—Nervous tissue makes up the brain, spinal cord and nervous system. Within a tissue, the cells may not be identical, but they still work together. For example, nervous tissue includes neurons, oligodendrocytes, astrocytes, and even more kinds of cells. The nervous system comprises vital body organs such as the brain, spinal cord and the supporting network of nerves and nervous tissue. The nervous tissue controls and regulates body functions. It comprises the neurons or transmitters of impulses and the neuroglia, the propagators of nerve impulses and providers of neuron nutrients. Nervous tissue comprises various nerve cells, each characterized by a long stem like part called the axon. In the nervous tissue, neurons are conducting cells that connect at synapses that act as switches, allowing information storage and processing. Glia (also called neuroglia) are support cells (functional and structural support) and they are involved in modulating neuron function. Diseases, like Parkinson’s disease, that affect nervous tissue functions, have serious consequences on the quality of life. The health of the tissue is affected adversely by a deficiency of dopamine (a neurotransmitter) and progressive death of brain cells. Health issues resulting from nervous tissue damage manifest in the form of rigid and unstable posture, and even death. Table 1 summarizes the dependence of tissue/organ function on tissue/organ microstructure while Figure 3 illustrates the cell microenvironment (niche) that directs cellular fate and function.

**Table 1:** Dependence of tissue/organ function on tissue/organ Microstructure.

Nanoscale	Microscale	Macroscale
support structures (<1 μm) to control individual cell behavior	support structures (1–100 μm) to control cell–cell interactions and cell-substrate interactions	support structures (>100 μm) for structural support
adhesion, migration proliferation	cell–ECM interaction	tissue–oran interaction

## 4. 3D Technology for Development of Tissue Engineering

### 4.1. 3D in Vitro Technology

Materials design for cell proliferation and differentiation is one of the key technologies for development of 3D *in vitro* culture systems. In conventional cell culture such as static tissue culture dish (2D), the initial rate of cell growth is higher, but the proliferation stop once the cells reached confluence. 3D structures have been investigated for the cell culture because they have larger surface for cell attachment and proliferation than 2D tissue culture dish and are preferable to assist the formation of 3D cell constructs which may resemble the structure and function of body tissues. In addition, 3D culture systems also play an important role in the substrate for *in vitro* cell culture to increase the number of cells as high as clinically applicable [29]. 3D materials that are able to regenerate or restore tissue and/or organs have begun to revolutionize medicine and biomedical science and have been used to support and promote the regeneration of tissues. The proliferation of cells in the 3D structures need an oxygen and nutrition supply. In this circumstance, the 3D materials should provide such an environment for cells living in close proximity. Diffusion of nutrients, bioactive factors, and oxygen through 3D materials is sufficient for the survival of large numbers of cells for extended periods of time. A major constraint of biodegradable polymeric materials for vascular tissue engineering is poor cell adhesion and lack of signals for new tissue generation. The presence of ECM within 3D structure is desirable for growth of stem cells and *in vitro* formation of remodeled vascular conduit [30]. In a study developed by Gingras M. *et al.*, axonal migration on a two-dimensional petri dish did not reflect the 3D environment *in vivo* [31]. A unique *in vitro* 3D model of motor nerve regeneration was developed to study motor neuron axonal migration and myelination. They explained that primary motor neurons were difficult to study in conventional culture systems because of their short-term survival without trophic support from glia. Therefore, mouse spinal cord motor neurons were seeded on a collagen sponge populated with Schwann cells and fibroblasts. This fibroblast-populated sponge was intended to mimic the connective tissue through which motor axons have to elongate *in vivo*. Addition of conventional neurotrophic supplements was not required for motor neuron survival but was necessary to promote deep neurite outgrowth. Additional works developed by the same authors have proved that a unique tissue-engineered model of peripheral nerve regeneration could be developed *in vitro* to study neurite outgrowth [32]. Mouse dorsal root ganglia neurons were seeded on a collagen sponge populated with human endothelial cells and/or human fibroblasts. Addition of nerve growth factor (NGF; 10 ng/mL) was not required for sensory neurons survival but was necessary to promote neurite outgrowth, as assessed by immunostaining of the 150 kDa neurofilament. A vigorous neurite elongation was detected inside the reconstructed tissue after 14 and 31 days of neurons culture, reaching up to 770 μm from day 14. Axons were often observed closely associated with the capillary-like tubes reconstructed in the model, in a similar pattern as in the human dermis. The presence of endothelial cells induced a significant increase of the neurite elongation after 14 days of culture. Besides the addition of NGF, axonal growth did not necessitate B27 supplement or glial cell co-culture to be promoted and stabilized for long-term culture. This indicates that the model might be a valuable tool to study the effect of various cells and/or attractive or repulsive molecules on neurite outgrowth *in vitro*. Horst *et al.*, developed new strategies in functional tissue engineering and drug delivery systems that involve rational design of biomimetic surfaces and 3D-hydrogel matrices [33]. They explained that their approach is to use modified 2D-surfaces or 3D-hydrogel matrices (native or synthetic polymers) to locally induce specific tissue regeneration especially in cases of impaired wound healing and nerve regeneration. The surfaces or matrices try to mimic key features of the native extracellular matrix and provide support for cell adhesion and growth, and are covalently modified by adhesion sequences or growth factors that target cell-type specific responses. Moreover, surface and matrices can be modified to release DNA-containing nanoparticles that transfect surrounding cells and induce specific protein production. In combination with shape giving (polymer-) surfaces, fibers or 3D-fibrous scaffolds, these matrices provide biological information combined with growth factor- or drug release inducing an endogenous healing response ideally resulting in the recovery of a diseased tissue. Table 2 compares 2D and 3D *in vitro* culture systems. Material design of scaffold for cell proliferation and differentiation is one of the key technologies for tissue engineering. Porous materials with 3D structures have been investigated for the cell scaffold because they have larger surface for cell attachment and proliferation than 2D culture dish and are preferable to assist the formation of 3D cell constructs which may resemble the structure and function of body tissues. In addition, the 3D scaffold also plays an important role in the substrate for *in vitro* cell culture to increase the number of cells as high as clinically applicable. In addition to the cell scaffold design, it is key for the cell culture in the 3D materials to develop the culture method suitable for cells proliferation and the control of their phenotype and biological functions. In the design of culture method, it is important to create a local environment suitable for cells cultured, such as oxygen, nutrient, and the metabolic environment as closely as possible to natural ones. The conventional static culture method does not always satisfy the requirement. The static medium cannot sufficiently supply oxygen and nutrition to cells in the 3D scaffold and exclude cell wastes for their proliferation and differentiation.

**Table 2:** Comparison between 2D and 3D *in vitro* culture systems.

2D Culture (Tissue Culture Dish)	3D Culture (Biomaterials)
The initial rate of cell growth is higher, but the proliferation stops once the cells reached confluence	Provides larger surface area available for cell attachment and spreading than that of 2D culture and can affect cell adhesion, spreading, and proliferation. Differentiation, cell viability and morphology of cultured cells in 3D biomaterials are significantly different with cells cultured in 2D tissue culture plate

### 4.2. Bioreactor Technology

The bioreactors for 3D tissues systems could be an advantageous alternative in terms of low contamination risk, easiness of handling and scaling-up. This equipment is specifically designed in order to provide a better control of the process as well as a safe and reproducible production of tissue construct. They can offer the technical means to perform controlled studies aimed at understanding the specific biological, chemical or physical effects. An important problem that has to be solved is the bioreactors scaling-up from laboratory scale to industrial level. This transposition requires the bioreactors to be specialized and adapted for a standardized production process.

Since a perfusion culture method facilitates the continuous exchange of medium and the constant removal of metabolic wastes, it has been used for several of cells [34,35,36,37,38,39]. Perfusion culture methods enhanced the viability and functions of murine osteosarcoma cells and bone marrow cells while it promoted matrix synthesis by chondrocytes cultured in a 3D collagen sponge and gave the cells better physiological conditions [40,41], resulting in their enhanced ability for secretion [42]. All the results suggest feasibility of the perfusion method in the cell attachment in the 3D scaffold. The property of scaffold material for cell attachment is one of the major factors contributing their morphology, proliferation, functions, and the subsequent tissue organization [43]. At first, cells attach to the material surface of a polymer scaffold, then spread, and proliferate. The 3D scaffold can provide larger surface area available for cell attachment and spreading than 2D tissue culture plate. Xie *et al.*, reported that the initial rate of cells grown was higher for the 2D culture, but once the cells reached confluent, their proliferation stopped [44]. However, the cells growth in the 3D scaffold was continued for longer time periods than that of 2D tissue culture plate. Other reports have demonstrated that cell proliferation was superior in the 3D scaffold than the 2D one [45,46]. When cells proliferate in a 3D scaffold by the conventional static method, the culture conditions often become worse with time in terms of oxygen and nutrition supply and wastes exclusion. In this circumstance, the perfusion of culture medium is one of the strategies to overcome the problem and to provide the culture conditions suitable for cell proliferation. As an advantage of the perfusion culture method, cells can receive the constant supply of nutrition and oxygen, while harmful metabolic products can be excluded from inside the 3D scaffold [47]. Since for the perfusion culture, the culture medium is continuously perfused to allow a continuous and optimal exchange of gases, the O_2_ partial pressure in the medium is considerably higher than for the conventional culture static method, and the desired pH value can be constantly stabilized over the entire culture period [48]. Besides, during the proliferation and differentiation of cells, metabolically active cells produce lactate into the medium, which would be harmful for cells due to the pH decrease. Although in most 2D culture methods, the lactate concentration is negligibly small, this is not the case in the 3D culture because of the high cell density. [49] By the perfusion method, the medium is recycled and permanently renewed. Since the level of lactate is kept at a physiological concentration, the cell damage is diminished. In this study, a higher level of osteogenic activity of stem cells in the collagen sponge reinforced with PGA fiber was observed by the perfusion method. This result can be explained by these features of perfusion culture. The enhanced viability and biosynthetic activity of cells by the perfusion method are consistent with other research results [50].

It is possible that slow perfusion does not improve the condition of mass transport throughout the sponge scaffold, resulting in suppression of MSC proliferation at the low flow rate. On the other hand, the higher perfusion rate results in an increase in the shear stress. This high shear stress may negatively affect the cells to suppress their proliferation. Cartmell *et al.*, revealed that the level of cell death within a scaffold increased with an increase in the perfusion rate [51]. As a result, the maximum proliferation would be observed at a middle flow rate of medium. Cartmell *et al.* reported that low medium perfusion flow rate (0.01 mL/min) promoted the proliferation of MC3T3-E1 osteoblast-like cells seeded on cylindrical human trabecular bone scaffolds, whereas a higher perfusion rate (0.2 mL/min) may have accelerated the differentiation of cells [51]. The flow rates used in this study (0.2–1.0 mL/min) are lower than those reported in other 3D perfusion methods (1.3–1.8 mL/min). It has been shown that a higher extent of differentiation in the perfusion bioreactor might be due to the suppressed cell proliferation. The lower medium perfusion flow rate (0.2 mL/min) did not induce MSC proliferation, whereas it accelerated the differentiation of MSC within the construct toward an osteoblast phenotype [52,53]. The medium perfusion flow rate showed a great effect on the MSC proliferation. Even if the flow rates had been matched, however, differences in the various perfusion experiments would result in different shear stresses applied to cells present in the scaffold. Although modeling the flow of medium through 3D porous scaffolds with complex microarchitecture to estimate local shear stresses is a challenging computational problem, such analyses are necessary to compare results between different perfusion experiments. Cell morphology and cell cycle may affect the extent of differentiation. Ma *et al.*, reported the effect of trophoblast differentiation on the non-woven fabrics with different porosities [54]. The differentiation depended on the cell morphology and the cell cycle. Other reports showed that there was a relation between the cell aggregation and the cell function [55]. The proposed technique of cell culture in 3D cell-scaffold constructs is based on the use of 3D fibrous scaffold to guide cell organization. In comparison with conventional culture, cells maintained in 3D culture more closely resemble the *in vivo* situation with regard to cell shape and cellular environment that can influence the behavior of cells [56].

**Figure 4:**
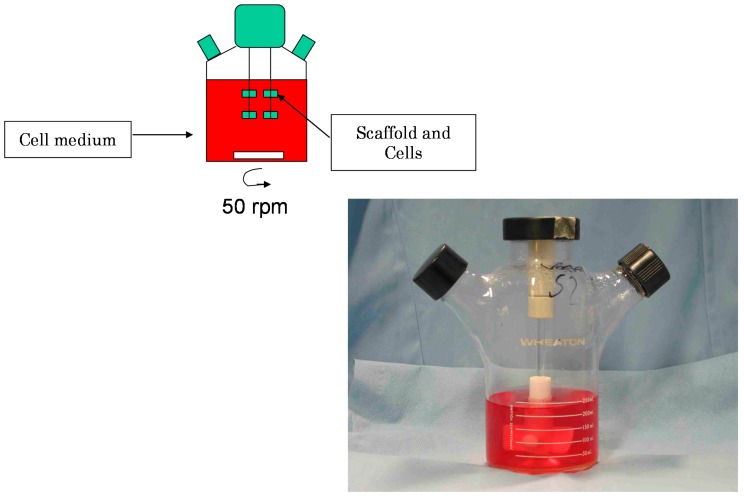
Schematic illustration of spinner bioreactor.

**Figure 5:**
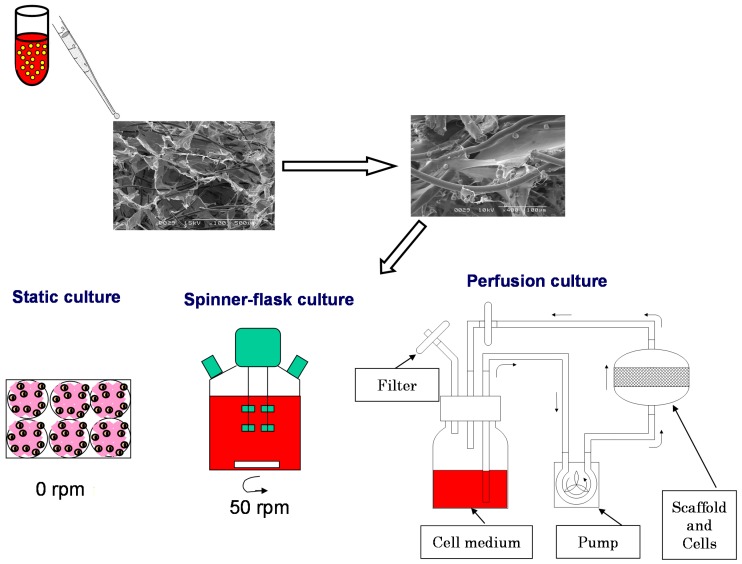
Principal of *in vitro* cell culture on 3D scaffolds in static, spinner-flask and perfusion culture.

There are other techniques used to generate 3D models, such as hanging drops/gravity culture plates, rotation culturing (e.g., spinner flasks) and spontaneous formed 3D cultures. Spinner-flask bioreactors have been used for the production of 3D tissue engineered biomaterials *in vitro* (Figure 4). The dynamic environment within bioreactors is known to significantly affect the growth and development of the tissue. Figure 5 shows principal of *in vitro* cell culture on 3D scaffolds in static, spinner-flask and perfusion culture.

### 4.3. 3D Printing Technology

**Figure 6:**
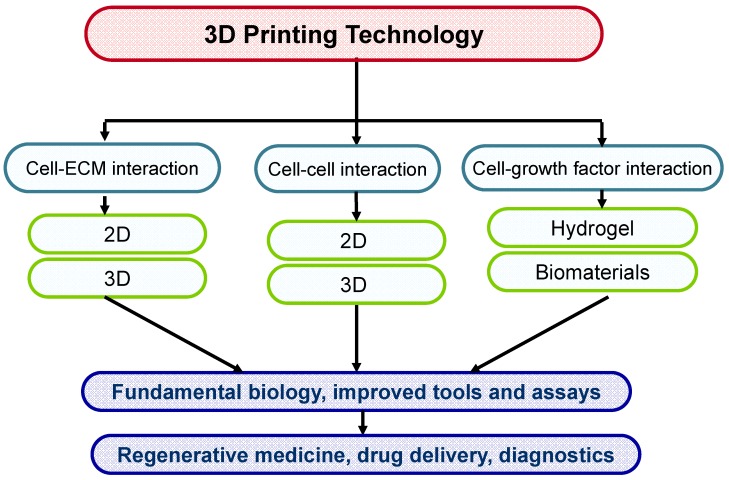
3D printing technology for tissue engineering applications.

3D tissue printing is the approach to print biological constructs in a small range from several millimeters to a centimeter, including several cell types and biomaterials at the same time. The latest technologies are trying to use 3D biomaterials printing and with cell patterning for constructing 3D scaffolds with living cells embedded in hydrogels. This technology may provide a better starting point for the cells by printing them directly on hydrogels and thus are faster to form functional tissue as compared to classical tissue engineering methods. To establish novel cell patterning techniques to control spatial location as well as cell-cell interactions, generating arrays of cells using biodegradable materials such as polyethylene glycol (PEG) or hyaluronic acid (HA) are very promising strategy to combine the cell patterning and microfluidics approaches to pattern cells in microfluidic channels. When it comes to patterning PEG hydrogels, typically two techniques are used, photolithography and capillary force lithography (CFL). In photolithography, a mask is used to selectively crosslink an underlying UV crosslinkable hydrogel. The unreacted part is easily washed with water and finally we can get successful patterns after developing. On the other hand, in CFL, a prepatterned polydimethylsiloxane (PDMS) stamp is placed on top of polymer surface directly after spin coating or spin casting. To provide mobility of the polymer, we can increase the temperature above glass transition temperature or if the polymer is mobile itself, it can spontaneously fills into the void space of the stamp. In usual organic polymers, organic solvents are used such as toluene or ethanol so that the contact angle is normally less than 90 degrees. In this case, the polymer takes up the void space according to the well known Young’s equation. In contrast, in most biological systems, the polymer is only soluble in water or PBS, or sometimes methanol or ethanol such that the contact angle is probably larger than 90 degrees. In this case, the polymer cannot rise into the void and recedes towards the bottom, which can be termed capillary depression. Figure 6 shows application of 3D printing in tissue engineering.

## 5. Development of Tissue Engineering in 3D Models

### 5.1. Potential of Biomaterials in Tissue Engineering

**Figure 7:**
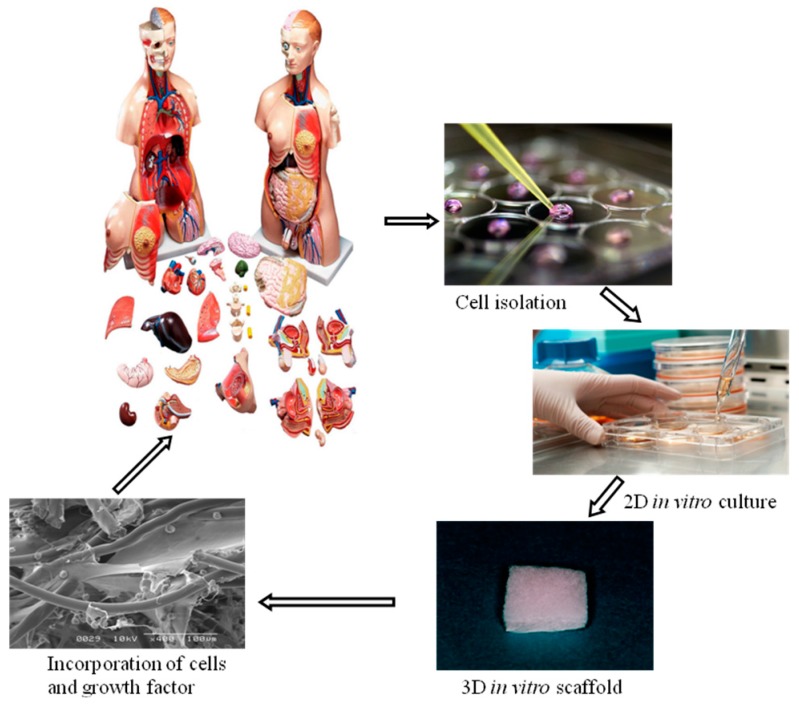
Schematic illustration of tissue engineering technology.

Tissue engineering is an interdisciplinary field that applies principles and methods of engineering toward the development of biological substitutes to improve the function of damaged tissue and organs (Figure 7). Its fundamental aim is the creation of natural tissue with the ability to restore missing organ or tissue function, which the organism has not been able to regenerate in physiological conditions. With that, it aspires to improve the health and quality of life for millions of people worldwide and to provide a solution to the present limitations: rejections, low quantity of donors, *etc.* The term regenerative medicine is often used synonymously with tissue engineering, although those involved in regenerative medicine place more emphasis on the use of stem cells to produce tissues. The motivation of using of tissue engineering in regenerative medicine is due to: (i) Since the 1970s, organ transplantation has become a common therapeutic approach for end-stage organ failure patients; (ii) Demand >> Supply (UNOS National Patient Waiting List); for example: 19,095 patients (1989), 80,766 patients (December 2002); (iii) Cost of organ replacement therapy: $305 billion (US, 2000). The interdisciplinary approach of tissue engineering is combinational technology of using molecular biology, materials engineering and reconstructive surgery. Tissue engineering needs scaffolds to serve as a substrate for seeding cells and as a physical support in order to guide the formation of the new tissue and is designed to regenerate natural tissues or to create biological substitutes for defective or lost organs by using cells [57,58,59]. There is no doubt that a sufficient supply of nutrients and oxygen to the transplanted cells is vital for their survival and functional maintenance. Mesenchymal stem cells (MSCs) have been known as one of the most used cells in tissue engineering and rapidly improved by several researches to evaluate their therapeutic applications. Every day, thousands of people of all ages are admitted to hospitals because of the malfunction of some vital organ. Because of a dearth of transplantable organs, many of these people will die. In short, the need for organs cannot be met by traditional methods of transplantation. Tissue engineering may change that. Tissue regeneration can be achieved by the following three key steps: Cell proliferation, cell seeding in a suitable scaffold, and the maintenance of the differentiation phenotype of the engineered tissues [1,60]. The property of 3D scaffolding biomaterials for cell attachment is one of the major factors contributing their morphology, proliferation, functions, and the subsequent tissue organization [45,54,61,62,63,64,65,66]. The last requisite is difficult to combine with the high porosity in volume of the material. Thus, it is necessary to use polymeric matrices with special or reinforced properties, especially if the polymer is a hydrogel.

**Figure 8:**
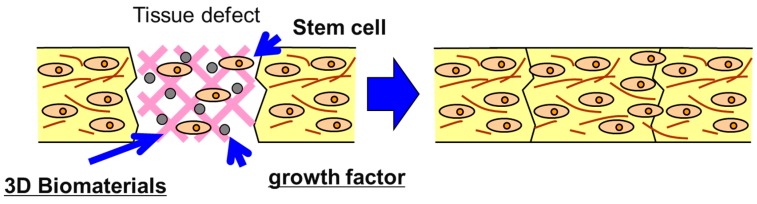
Schematic illustration of tissue engineering based on 3D biomaterials technology.

Biomaterials technology is one newly emerging biomedical form to create new device and induce the regeneration of detective and injured body tissues and organs as well as to substitute the biological functions of damaged organs. To this end, the cells of high proliferation and differentiation potentials are being combined with some cell scaffolds and the biological signals of growth factors and genes to regenerate tissue. Figure 8 illustrates the potential of biomaterials in tissue engineering applications. Since there are some cases in which cells are genetically innovated to produce the growth factors inducible angiogenesis and tissue regeneration, the technology of gene delivery is also necessary for tissue engineering. Current developments in the technological fields of biomedical and tissue engineering, bioengineering, biomechanics, microfabrication and microfluidics have led to highly complex and pertinent new tools for *in vitro* and *in vivo* applications. The purpose of biomaterials technology is to mimic organ tissues *in vitro* in order to partially reduce the amount of *in vivo* testing. These types of systems can enhance functionality of cells by mimicking the tissue architecture complexities when compared to *in vitro* analysis but at the same time present a more rapid and simple process when compared to *in vivo* testing procedures. The development of new technology for analysis of engineered tissues can be achieved through the combination of these research domains. Combining these advanced research domains, we then present a new area of technology that allows analysis *in vitro* on engineered tissues. An extension of the biomaterials technology has also allowed tissue and organ development, which can be considered as a first step towards the replacement of animal testing using a combined organ model. Recent advances have been in exploring materials which are not passive and walled off by the body but actively participate in the body’s efforts to repair itself. Such biomimetic and bioactive materials are designed to more accurately mimic the body’s natural structures and functions from macro to micro- to nano-levels. Materials capable of exhibiting biorecognition events have begun to revolutionize medicine and biomedical sciences by providing tools for inducing or probing biomolecular and cellular interactions. Biomedical materials may mimic properties of the biological milieu and in particular, are promising for creating bioactive materials for a variety of applications ranging from tissue engineering to microdevices. The most common strategy to produce bioactive materials has been to bind peptides, proteins, nucleic acids, or other biological molecules directly to a synthetic or inert surface, structure, or polymeric molecule. However, simple and robust methods of functionalizing these materials with multiple biomolecules would be a critical advance for widespread application of these materials. Therefore, it is necessary to synthesize and characterize robust multifunctional materials containing immobilized biomolecules that can be used to functionalize materials with a variety of biological molecules. Specifically, it is important to utilize the high binding affinity and specificity of the biomolecules bonding to conjugate functionally active biomolecules to biomaterials. The conjugation of biomolecules to materials can be used to generate multi-functional biomaterials that can, with high specificity, bind or “capture” a plethora of biotinylated biomolecules. In addition, it is important to test the utility of these materials by generating gradients of biomolecules within materials and analyze their ability to influence cell function. Biomedical materials are useful for many biomedical applications such as high throughput drug screening and biosensing assays, as thin films for coating medical devices, or as scaffolds for tissue engineering. In medical diagnostics and therapeutics there is a continuous effort to enhance methods, materials, and devices to improve patient care. In recent years, the development of novel biomaterials and their application to medical problems have dramatically enhanced the treatment of many diseases [67,68]. Biomaterials such as polymers, ceramics and metals have been used for several decades for medical applications. In addition to the over 40,000 pharmaceutical preparations in use, it is estimated that currently there are over 8000 medical devices and 2500 diagnostic products that employ biomaterials. Despite the widespread use of materials in medicine, many biomaterials lack the desired functional properties for stimulating a specific biological response and have not been engineered for optimized performance. Therefore, there is an increasing need to develop new materials to address such problems in medicine and biology.

### 5.2. Advanced Technology behind 3D Biomaterials

Biomaterials and the use of new materials underpin the race for tissue-engineered products because of the strong biological activity that current researches appear to indicate for these materials. There is no doubt that tissue engineering using biomaterials will be the novel innovation of the future. Remarkably, the recent identification of nanotechnology with enhanced ability to mimic natural ECM proteins such as collagen structure has led to the discovery of a class of biomaterials with specific properties for tissue engineering applications and regenerative medicine therapy. The design of materials that can regulate cell behavior such as proliferation and differentiation is a key component for the fabrication of 3D tissue-engineered scaffolds. From the viewpoint of immune system response of the body, the implanted biomaterials should mimic the structure and biological function of native extracellular matrix (ECM), both in terms of chemical composition and physical properties. Therefore, in order to mimic the biological function of ECM proteins, the scaffold materials used in tissue engineering need to be chemically functionalized to provide appropriate niches for cell proliferation and differentiation and ultimately tissue regeneration as ECM does. Nano-engineering of molecular templates and supra-molecular structures has been used to engineer new structure of 3D biomaterials [69]. On the other hand, self-assembling amphiphilic peptide and protein systems that self-assemble to form various nanostructures like nanofibers, nanotubes, vesicles, helical ribbons and fibrous scaffolds [70,71]. These artificial proteins can form hydrogels in response to pH and environmental changes. Protein hydrogels can be used for advanced wound closure and tissue repair in regenerative medicine and tissue engineering [59]. Targeted tissue engineering using ligand-receptors biomaterials are very attractive technology that are based chemically modified biomaterials to mimic ECM to direct stem cells differentiation [61]. Controlled release technology of growth factors and gene therapy technology from implanted 3D biomaterials are also alternative methods to regenerate new tissues [72,73,74]. Table 3 summarizes advances in 3D biomaterials for tissue engineering applications.

**Table 3:** Advances in 3D biomaterials for tissue engineering applications.

Advances in biomaterials for tissue engineering applications.	Nano-engineering of molecular templates and supra-molecular structures to engineer new structure of 3D biomaterials [69].
	Self-assembling amphiphilic peptide and protein systems that self-assemble to form various nanostructures like nanofibers, nanotubes, vesicles, helical ribbons and fibrous scaffolds [72].
	Artificial proteins that self-assemble to form hydrogels in response to pH and environmental changes. Protein hydrogels can be used for advanced wound closure and tissue repair in regenerative medicine and tissue engineering [28].
	Used as scaffolds to fabricate nanowires, templates for metallization (Ex: Histidine-rich peptide nanotubes were metallized with gold nanocrystals and the organic peptide scaffold was removed to make a conducting gold nanowires [66].
	Targeted tissue engineering using ligand-receptors biomaterials that are chemically modified to mimic ECM to direct stem cells differentiation [30].
	Combinational technology of micro- and nano-fabrication to mimic mechanical properties of ECM to accelerate tissue regeneration [65].
	Controlled release technology of growth factors from implanted 3D biomaterials to regenerate new tissues [25].
	Gene therapy technology in combination with 3D biomaterials for tissue engineering applications [65]
	Enhancement of angiogenesis by using 3D biomaterials to enhance survival of transplanted stem cells [4].

## 6. Future Prospects

The increasing interest in 3D *in vitro* technology has stimulated researchers to scrutinize biological elements and learn from nature. The topic discussed above regarding materials technology give us a wide knowledge about the basic principle underlying 3D *in vitro* systems. It gives a general overview of different kinds of culture systems. The elucidation of the above goals will open many doors and lead to significant improvements in biological tools, drug discovery process, lead identification as well as therapeutic approaches. The miniaturization of this approach allows one to perform many more experiments than previously possible in a simpler manner. 3D *in vitro* technology aim to develop a set of tools that are simple, inexpensive, portable and robust that could be commercialized and used in various fields of biomedical sciences such as drug discovery, diagnostic tools, and therapeutic approaches in regenerative medicine. Throughout the chapters discussed above, we inform the reader about the potential applications of different 3D *in vitro* systems that can be applied for fabricating wider range of novel biomaterials for the use in biotechnology. With appropriate references and examples, we aim to open up the reader’s mind, incorporating a wider range of knowledge about 3D *in vitro* systems. These specific structures have inspired the researchers to use them in various areas of science like biotechnology, nanotechnology and medicine. In this article, a few applications of biomaterials in regenerative medicine are discussed. Biotechnology promises numerous micro- and nano-scale 3D systems which can act as bio-mimicking systems. Research conducted worldwide will result in the discovery of newer and smarter biomaterials to treat various kinds of diseases. Even though there are certain technological hurdles, these can be overcome by understanding the drawbacks of the individual technology and finding an alternative to overcome these drawbacks. Thus, 3D systems and their versatile applications have kindled the interests of numerous scientists and researchers to find newer ways to apply it in the field tissue engineering. Biomaterials are evolving rapidly with much more potential impacts in treatment of diseases like cancer, diabetes, respiratory diseases like asthma, ocular diseases, to mention a few.

Numerous studies are being conducted worldwide to discover newer solutions that can be effectively applied to treat these diseases using stem cells technology. Results obtained in biotechnology are inspiring the scientific community to discover new innovative non-invasive tools at the micro- and nano-scale level for such purposes.
